# Analysis of the quality of remote working experience: a speech-based approach

**DOI:** 10.1007/s41233-022-00049-w

**Published:** 2022-03-30

**Authors:** Simone Porcu, Alessandro Floris, Luigi Atzori

**Affiliations:** 1 Department of Electrical and Electronic Engineering, University of Cagliari, Piazza d’Armi, Cagliari, 09123 Italy; 2 CNIT, University of Cagliari, Piazza d’Armi, Cagliari, 09123 Italy

**Keywords:** Quality of experience, Speech analysis, Voice over IP, Remote working, ANOVA

## Abstract

The current pandemic situation has led to an extraordinary increase in *remote working* activities all over the world. In this paper, we conducted a research study with the aim to investigate the Quality of Remote Working Experience (QRWE) of workers when conducting remote working activities and to analyse its correlation with implicit emotion responses estimated from the speech of video-calls or discussions with people in the same room. We implemented a system that captures the audio when the worker is talking and extracts and stores several speech features. A subjective assessment has been conducted, using this tool, which involved 12 people that were asked to provide feedback on the QRWE and assess their sentiment polarity during their daily remote working hours. ANOVA results suggest that speech features may be potentially observed to infer the QRWE and the sentiment polarity of the speaker. Indeed, we have also found that the perceived QRWE and polarity are strongly related.

## Introduction

It is a matter of fact that the current pandemic situation has led to an extraordinary increase in remote working (also referred to as teleworking or telecommuting) activities all over the world. Accordingly, telecommunications services have become even more important than in the past to interact with our colleagues during the lockdown period; additionally, these have been suddenly vital to maintain alive our relationship with our parents, relatives and friends. For these reasons, Voice over IP (VoIP) services, such as Skype, Teams, Zoom, Google Meet, have taken a major role in our life during the last year. The utilization of remote working as an alternative mode of work is not novel; however, prior to the COVID-19 pandemic this mode was only optionally chosen by some businesses or single workers and was not widespread. Instead, the COVID-19 pandemic has required millions of workers an obligatory shifting to remote working, thus becoming predominant with respect to the *normal* office-based mode characterized by *real* (i.e., physical) interactions with our colleagues. As this new setting is expected to become the *new normality*, there is the need to investigate the experience perceived by the remote workers.

Common quality evaluation approaches related to the remote working context imply the recruitment of a group of workers and the conduction of well-defined interviews with the aim to identify the advantages and drawbacks of the working context based on the perceived worker’s experience [1, 2]. However, in this paper we aim to research on the possibility to apply a different evaluation methodology inspired by the concept of affective computing, which concerns the analysis of human emotional variables naturally revealed during the user-system interaction. Affective computing research typically investigates the relationship between the human perceived quality (Quality of Experience, QoE) and affective behaviors driven by human emotions, which can be automatically inferred from facial expressions, speech, and body gestures [3–5]. In particular, we focus on the speech signal for two main reasons: speech is one of the most natural ways for humans to express their emotions; and relevant Speech Emotion Recognition (SER) research has identified speech features that are indicative of different emotions [6, 7].

We then define the quality of remote working experience (QRWE) as the quality perceived by the worker when conducting remote working activities. The perceived quality includes the influence of the potential negative side of remote working, namely, work-home interference, ineffective communication, procrastination, and loneliness [1, 2]. A clear understanding of the QRWE by the employees is of vital importance for the employer as this can highlight limits and potentials of the adopted remote working settings. It may result that low-quality communications due to poor network conditions may become frustrating for the employees or that working at home with interactions with other family members can be disturbing for the perceived level of experience. With this knowledge it would then be possible to take corrective actions to improve the overall team productivity. Also, these actions can be personalized if it was possible to estimate the quality for each individual employee. To this end, the mentioned method for QRWE estimation based on implicit emotion recognition through speech-based features would be of great help.

On the basis of these considerations, we conducted a research study with the aim to investigate the QRWE and to analyse its correlation with implicit emotion responses estimated from the speech of video-calls or discussions with people in the same room (colleagues or family members). To this end, we implemented a software that captures the audio when the worker is talking and extracts and stores several speech features. This tool has been used to conduct subjective tests that involved 12 people that were asked to provide feedback on the quality and assess their polarity during their daily remote working hours. We then conducted an ANOVA analysis to understand the correlation between the subject provided feedback and the extracted speech features. ANOVA results suggest that speech features may be potentially observed to infer the QRWE and the sentiment polarity of the speaker. Indeed, we have also found that the perceived QRWE and polarity are strongly related. In particular, positive quality perceptions are related to neutral and positive polarity whereas low quality is related to negative emotions. These results lay the groundwork for conducting further research towards the creation of an estimator of QRWE and polarity based on speech features.

The paper is structured as follows. Section “Related work” discusses related work. Section “Proposed methodology” presents the proposed research methodology. In section “ANOVA analysis”, we discuss the results of the ANOVA analysis. Section “Discussion” highlights the findings concerning the QRWE. Finally, Section “Conclusion” concludes the paper.

## Related work

The research study considered in this paper deals with Speech Emotion Recognition (SER) and affective computing. Speech is one of the natural ways for humans to express their emotions and the SER research studies search for speech features that are indicative of different emotions [6, 7]. Although researchers still have not found the optimal feature set for the SER process, the reviews in [6, 8] identify the vocal tract features and the prosodic features as the speech features more related to the emotions. Indeed, the former are responsible for producing different sound units in different emotions whereas the latter makes human speech natural including duration, intonation and intensity. Spectral features, e.g., the MFCCs (Mel frequency cepstral coefficients), are the common features derived from the cepstral domain that represent vocal tract information, while the prosody is represented by acoustic features, such as pitch frequency features, duration and energy-related features. In [7], these features are referred to as Low-Level Descriptors (LLD).

The affective computing concerns the analysis of human emotional variables naturally revealed during the user-system interaction and investigates the relationship between the perceived quality (QoE) and affective behaviors driven by human emotions, which can be automatically inferred from facial expressions, speech, and body gestures [3]. There are several studies in the literature focused on the relationship between psychophysiological responses (e.g., electroencephalography (EEG)) and QoE [9]. For example, affective brain-computer interfaces (BCI) are utilized in [10] to measure the user’s emotions when distorted speech is listened. Results have shown that features extracted from the EEG-based BCI could improve the accuracy of objective QoE models. The main drawback of these methods is that they are very invasive for the users and cannot be used in the real life.

To the best of authors’ knowledge, the research in [3] is the first study that hypothesizes that the QoE perceived in voice communication is correlated to the affective behavior, which will vary across networking conditions. Three types of affective speech features are considered: acoustic, lexical and discourse. Subjective experiments are conducted to examine how QoE changes when communication quality is impaired by different network QoS conditions, i.e., delay, bandwidth and loss rate. The proposed approach is evaluated by using classification techniques based on Support Vector Machine (SVM) and k-Nearest Neighbour (kNN). Results have shown that acoustic features alone provide more accurate QoE prediction than lexical and discourse features. However, the aggregation of all features slightly improves the performance. A similar study is proposed in [11], where users emotional behaviors were also considered, in addition to vocal and lexical features, to train the SVM-based prediction model. Through subjective experiments the vocal and emotional behaviors of the participants, along with the network conditions and their perceived QoE, were recorded. Also in this case, the best accuracy is obtained when all features are combined. However, the vocal feature set alone achieved good accuracy.

When considering video services, affect-based QoE evaluation is driven by user’s facial expressions. In [5], the relationship between speaker and listener’s facial expressions and the quality of the presentations was analyzed. Emotion was tracked through Zoom face video snapshots using facial emotion recognition. A score was given after each presentation by all participants except the presenter and it was found that the happier the speaker is, the happier and less neutral the audience is. Also, the presentations that triggered wide swings in “fear” and “joy” among the participants are correlated with a higher rating. In [12], the prediction of user’s QoE for video services is based on both user’s face expressions and network QoS parameters. Different Machine Learning (ML) algorithms are employed to test the system but the greatest prediction accuracy is achieved with gradient based-boosting and Random Forest bagging based methods. In [4], we have already investigated the design of a ML-based QoE prediction model for video services by considering human facial expressions and gaze direction as the training features. We conducted two different experiments: i) a crowdsourcing test in which participants were asked to watch and rate the quality of videos subject to buffering-related events; ii) a laboratory test in which participants were asked to watch and rate the quality of videos subject to blurring impairments. The facial metrics and the respective QoE values provided by the participants were used to train different ML classifiers aimed at QoE estimation.

In this paper, we focus on the evaluation of the QRWE based on implicit emotion recognition through speech features. In particular, we consider the LLD features, which are demonstrated to be linked to human emotions. The objective is to investigate the correlation between the perceived QRWE and implicit emotion responses estimated from the speech of video-calls or discussions with people in the same room when conducting remote working activities.

## Proposed methodology

The research objective considered in this paper is the study of the QRWE, which we define as the quality perceived by the worker when conducting remote working activities, which relies on people’s speech analyses. In the following subsections, we describe: the considered setting of remote working, the objective of the study, the developed system for the proposed analysis, the features extracted for the prediction of the quality, and the performed data collection and preprocessing.

### Remote working

Remote working is defined as “working outside the conventional workplace and communicating with it by way of telecommunications or computer-based technology” [13]. In this study, we partially rely on this definition as in the scenarios we consider the people may be working in the conventional workplace but without having traditional physical interactions with other colleagues. Specifically, we consider as *remote working* the new modality of conducting our working activities, which is becoming predominant (especially as a consequence of the physical social interaction limitations imposed by the pandemic) with respect to the *normal* office-based modality where we had frequent *real* (i.e., physical) interactions with our colleagues. During remote working activities, we are most often physically isolated from our colleagues, i.e., we do not share rooms with other people except at home if we work remotely or at the workplace if safety guidelines requested by the COVID-19 pandemic are strictly respected (e.g., large office room, limited number of workers, plentiful ventilation, and frequent cleaning). We consider even working at the workplace as remote working because during this pandemic period physical meetings are strongly discouraged and the majority of activities occur remotely through the Internet, which results in a significant amount of our time spent on audio-video calls. As this new setting is expected to become the *new normality*, there is the need to investigate the experience perceived by the “Remote Workers” and how it is affected by the different influencing factors. There are some potential negative effects that have been identified in the remote work context, i.e., work-home interference, ineffective communication, procrastination, and loneliness [1, 2]. For example, working at home means more interruptions from family members, which may negatively influence work effectiveness. Moreover, online communication may not be perceived as efficient as face-to-face communications in the office and may also be impaired by low-quality Internet connection.

### Objective of the study

Therefore, the objective of this study is to investigate the QRWE by asking colleagues from the University involved in different jobs, such as secretary, researcher, and PhD students, to provide their feedback related to the perceived remote working quality. To evaluate the perceived QRWE, the workers were asked to consider the multitude of influence factors that impacted (positively or negatively) on their remote working activities. These could involve system factors (e.g., slow Internet connection, low quality of the audio/video signals), ambient and context factors (e.g., lighting conditions, noisiness, work-home interference, comfort), and psychological factors (e.g., loneliness, isolation, sadness, frustration).

Other than evaluating the quality of remote working activities, we also aim to investigate whether the affective behaviors from the speech (captured when the worker is engaged in voice communication, such as video-calls or discussions with people in the same room) may provide insights concerning the perceived QRWE. To this end, we implemented a software that captures the audio signal when the worker is talking and extracts several emotion-related speech features. The software also asks periodically the worker to provide a feedback regarding the perceived QRWE of the working experience.

The remote working scenario considered in this study sees the worker working in a desk with his Personal Device (PD), such as a PC, a laptop or a tablet. The working activity we focused on is the Web call. However, we have also considered the cases of working room shared with a colleague (at the office) or with a relative/roommate (at home). Therefore, the software can be (automatically) activated for three different cases: i) the worker talks during a Web call; ii) the worker talks with a colleague in a shared office; iii) the worker talks with a relative/roommate at home.

### The developed system for the analysis

**Fig. 1:**
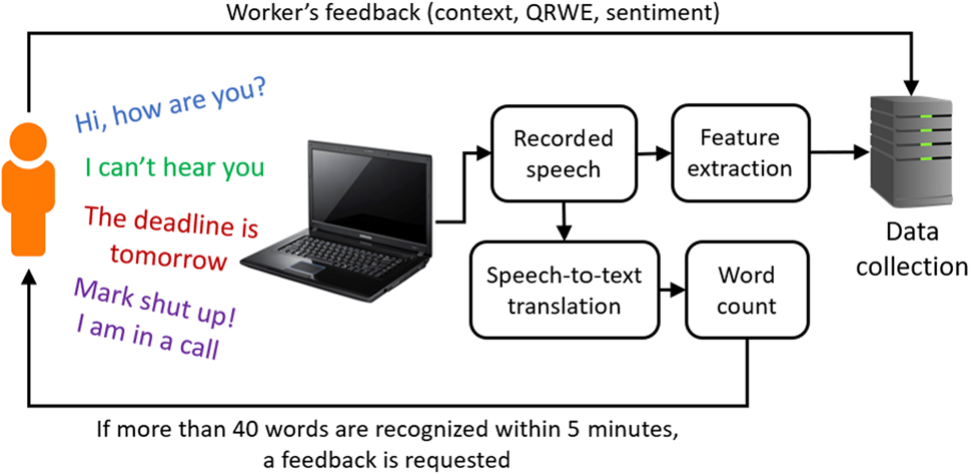
Framework of the proposed methodology

To collect data, we designed and implemented a software system that runs in background while the worker is working with her PD, and automatically listens when the worker is talking to collect data. In Fig. 1, we show the framework illustrating the proposed methodology. Even if there are commercial tools that could be used to record the audio and extract the needed features, we had to develop an ad hoc solution as we had specific needs in terms of operations to be performed to achieve the desired framework, as it will be clarified in the following.

**Fig. 2:**
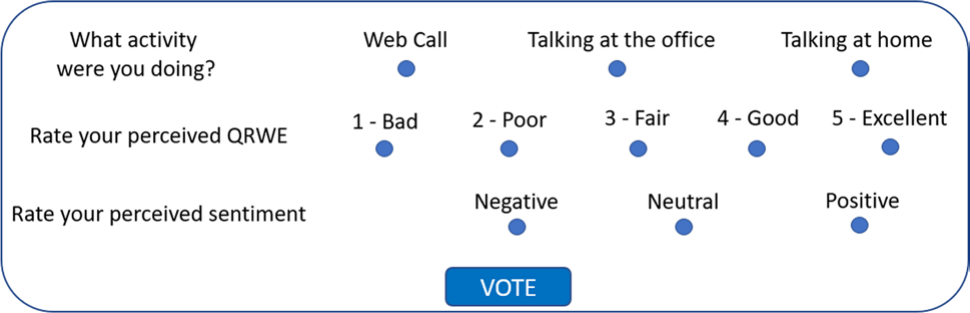
Rating window that appears when a feedback is requested to the worker

After starting it, the software keeps running in background capturing the audio from the microphone of the PD. The action of listening is computed by the Vosk offline open source speech recognition toolkit.^1^ This module is provided with different n-gram language models encoded as weighted finite-state transducers (FSTs). For our experiment, we used the Italian Language model because experiment participants were all Italian. However, different language models are available to repeat the experiment with people from other countries. When a speech is detected, it is analyzed in real-time by two subsequent processes. The first process converts the recorded speech into an array of text strings, where each string is a recognized word. The second process extracts several features from the recorded speech, which are discussed in Section “Features”. After these two processes are concluded, the recorded speech is discarded. The array of strings, in turn, undergoes the word count process, which counts the total number of words contained in the array of strings. This count is stored in a variable to check the condition for feedback request, which will be explained later. After this process is concluded, the array of strings is also discarded. By discarding both the recorded speech and the corresponding translated text, the privacy of the workers is protected. Indeed, only the extracted speech features are stored, from which the speech of the worker cannot be reconstructed.

With regard to the worker’s feedback, the software works as follows. The software counts the words stored within consecutive 5-minute-long time windows. If the number of words counted within 5 minutes is greater than 40, the worker is supposed to be talking with someone and feedback is requested. In Fig. 2, we show the feedback window that appeared in front of the worker when the 40-words condition within 5 minutes was respected. First, to define the context, the worker was asked to select the type of activity he/she was carrying on; as the feedback was requested when some speech was acquired, it is supposed the worker was talking with someone, which can happen on the Internet during a Web call, or with people in the same room, such as relatives, roommates, friends or colleagues. The perceived QRWE could be rated in terms of the Absolute Category Rating (ACR) scores defined by the ITU Rec. P.800, which are widely used to evaluate the QoE. The ACR scores are 5, i.e., 5 (Excellent), 4 (Good), 3 (Fair), 2 (Poor), 1 (Bad) [14]. The workers were carefully instructed regarding the meaning of the quality scores before participating in the subjective experiment. Finally, the perceived sentiment polarity is asked, which can be expressed as positive, negative and neutral [15].

The software is implemented with the Python language and it is a multi-thread software based on a shared queue that collects the speech sequences by recording at the default sample rating set by the PD’s microphone. The features are extracted every time the worker speaks. In order not to miss any sentence or pronounced word, the software executes two parallel different threads: the listening thread and the features extraction thread. Whenever the latter thread starts, it clears the RAM by discarding the recorded audio from the queue (RAM-usage lower than 800 MB).

### Features

We considered spectral and acoustic (energy-related features) speech features because they are the most used for SER [7, 16, 17]. For example, the combined Spectrogram-MFCC model in [7] results in an overall emotion detection accuracy of 73.1%, outperforming state-of-the-art methods whereas acoustic features are used in [3] for QoE prediction.

The considered features are: MFCCs, Chroma, and Mel Spectrogram. The MFCCs describe the overall shape of a spectral envelope by computing the discrete cosine transform (DCT) of the real logarithm of the short-term energy displayed on the Mel frequency scale. The MFFCs consist of an array of 40 log-energies. However, with regard to SER tasks, state-of-the-art studies usually consider only the first 12-13 MFCCs, which are sufficient to analyze the vocal features. Nevertheless, we considered a subset of 20 MFCCs because our objective is to investigate the relationship between these features and the QRWE. The Chroma is a 12-element feature vector that identifies how much energy of each pitch class (e.g., C, C#, D, D#, E, B), is present in the speech signal. Finally, the Mel Spectrogram is composed of the Mel Scale and Spectrogram. The Mel Scale is the result of non-linear transformations of the frequency scale. The Spectogram transforms frequency to log scale and the amplitude to decibels. Combining the Spectogram with the Mel Scale we obtain the Mel Spectogram, which partitions the Hz scale into slices, and transforms each slice into a corresponding slice in the Mel Scale. We considered 128 Mel features for our experimental tests.

### Data collection and preprocessing

To collect data, we asked 12 people (colleagues and students from the University) to participate to a subjective test and to use our software during a remote working week. All participants (3 females, 9 males, age range: 25–45 years) have no speaking or hearing issue, speak the same language (Italian) and the same dialect (Sardinian) and have the same cultural background (Sardinian). These people were carefully instructed before using the software and were all trustworthy and committed to conduct the tests by exactly following the given instructions. Specifically, they were said to consider and rate the QRWE perceived during the 5 minutes of remote working activities preceding the feedback window appearance. Also, they had to use headphones during Web Calls so that the PD’s microphone would not ‘hear’ other voices besides the participant’s voice. Moreover, we assured participants that their privacy was protected and their recorded speech could not be reconstructed. Then, they were free to talk about every subject as if they were not monitored. The assessment lasted for a week and each working day the participants had to have the software open for the full working day.

The software was set to save automatically into a Comma Separated Values (CSV) file the data regarding the extracted speech features (as described in section “Features”) and the timestamp. Specifically, for each listened sentence, 20 MFCCs features, 12 Chroma features, and 128 Mel features were saved. Moreover, when the user filled the requested feedback, the answers were saved, together with the timestamp, into a second CSV file. By matching the timestamps saved for both the feedback and the speech features, we were able to identify to which features (5-minute-long set of features preceding the feedback’s timestamp) the feedback referred to.

**Fig. 3:**
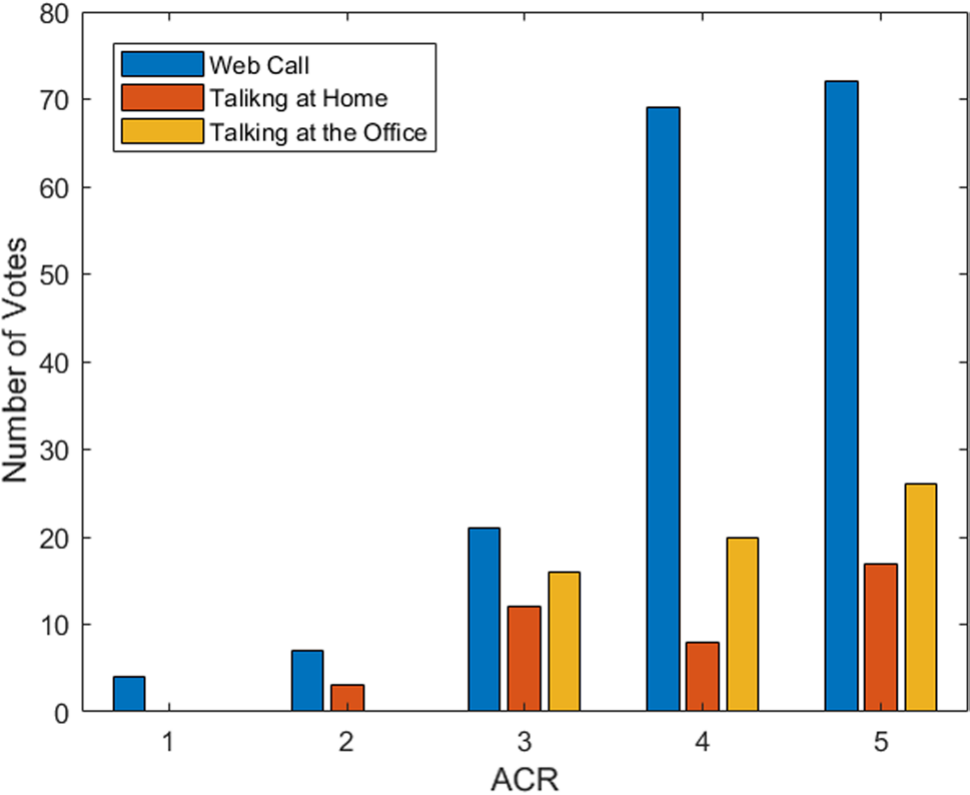
Number of the ACR rates provided by workers for each of the considered working categories

In total, we collected 275 feature samples and feedback samples. Each sample is related to the 5-minute time window during which the subject said at least 40 words. Each feature sample is composed of 160 speech features whereas each feedback sample is composed of the working activity, the perceived QRWE and the perceived sentiment polarity (Fig. 2). Note that there are no collisions among different activities, i.e., there are no recorded samples regarding, for example, having an online meeting and interacting briefly with some family members. But each sample only regards one specific working activity. The working activities are distinguished in 3 categories concerning the three ‘talking cases’ considered in the remote working scenario described in section “Remote working”, namely, “Web Call”, “Talking at the office”, and “Talking at home”. Among the 3 working activities, 173 samples were related to “Web Call”, 62 to “Talking at the office” and 40 to “Talking at home”. Fig. 3 shows the number of the ACR rates provided by workers for each of the considered working categories. It can be seen that for “Web Call” the whole ACR scale was used by participants whereas for “Talking at home” and “Talking at the office” only the greatest 4 and the greatest 3 ACR values were used, respectively. However, it must be considered that the number of ACR rates provided for “Web Call” was almost 3 times that of “Talking at the office” and more than 4 times that of “Talking at home”. Therefore, “Web Call” was the most frequent activity during remote working. The reason is that, as expected, most of the time spent during remote working regarded Web audio-video calls. Talking at the office with colleagues was the second most frequent case while the least frequent case concerned talking with relative/roommate at home.

**Fig. 4:**
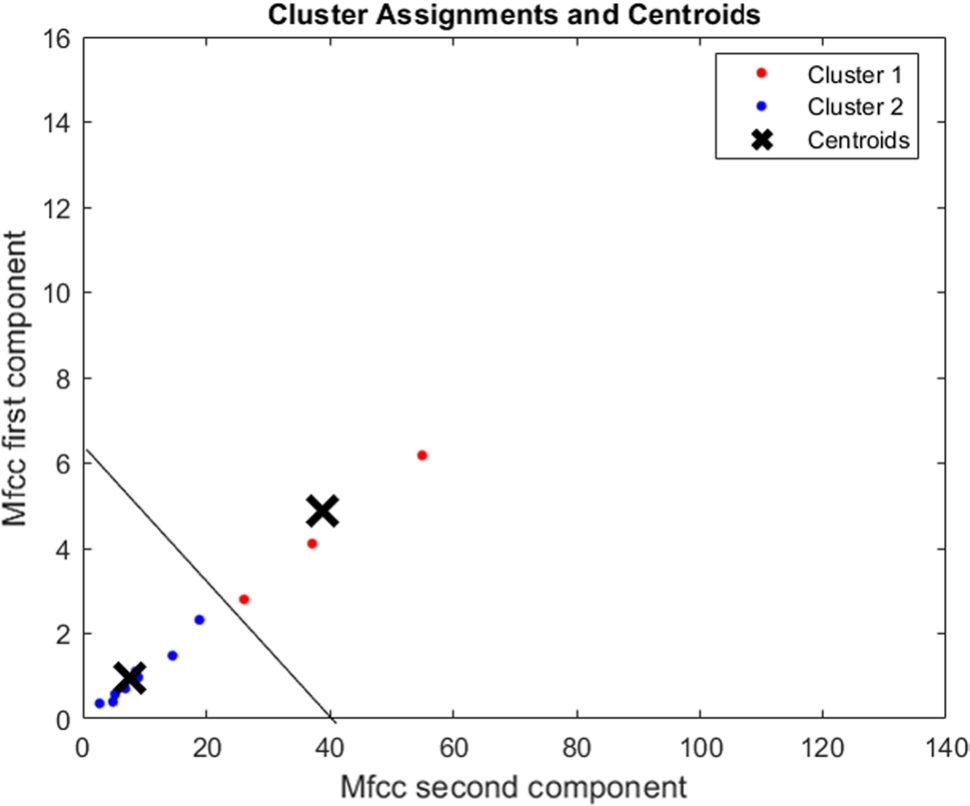
Example of clustering analysis to identify different people from MFCCs features

Since the microphone could record the voice of more than one person during the “Talking at the office” and “Talking at home” cases, we performed a preliminary statistical analysis to investigate whether the features belonging to different people could be identified. To this end, we performed a clustering analysis on the MFCCs features using the K-means clustering algorithm. Indeed, the MFCCs are typically used for speaker recognition tasks [18]. We performed the clustering analysis for each sample collected for each participant. As said before, it is important to note that we asked the participants to use the headphones during Web calls to avoid to capture other voices (from other people participating to the call) by the PD’s microphone. Therefore, in this case we were sure to analyze the speech of the participant and we used the features extracted for Web calls as the ground-truth. Then, we performed the clustering analysis for the other 2 cases (“Talking at the office” and “Talking at home”) obtaining 2 different clusters of features. By comparing these clusters with the ground-truth cluster, we were able to identify the features belonging to the participant and to keep them as well as to discard the other features (supposed to belong to other people talking during the test). Figure 4 shows an example of clustering analysis on a sample where two people were recorded while talking at home. It can be seen as the features belonging to different people are categorized into different clusters. In particular, the Cluster1 contains 4 samples of features belonging to external people whereas the Cluster2 contains 30 sets of samples belonging to the test participant.

## ANOVA analysis

In this section, we present the results of the data analysis using the one-way analysis of variance (ANOVA) and multiple comparison techniques. The objective is to investigate whether different perceptions of the remote workers, in terms of ACR score and sentiment polarity, are correlated to different distributions of the collected speech features.

**Table 1:** ANOVA test for ACR scores and speech features

Activity	All features		MFCC features		Chroma features		Mel features	
	F score	p value	F score	p value	F score	p value	F score	p value
Web Call	195.37	$< 0.001$	48.12	$< 0.001$	23.24	$< 0.001$	193.44	$< 0.001$
Talking at office	81.32	$< 0.001$	37.07	$< 0.001$	39.47	$< 0.001$	97.70	$< 0.001$
Talking at home	53.55	$< 0.001$	10.02	$< 0.001$	9.95	$< 0.001$	57.78	$< 0.001$
All activities	288.74	$< 0.001$	70.13	$< 0.001$	25.61	$< 0.001$	286.22	$< 0.001$

### Preprocessing of speech features

As described in section “Proposed methodology”, from the data collection we obtained 275 5-minute long speech feature samples. Each of these samples contains, for each listened sentence, 20 MFCCs features, 12 Chroma features, and 128 Mel features. It was then needed to preprocess these features to obtain a scalar value that could be used (together with the ACR score provided by the worker and related to the collected speech feature sample) to compute the ANOVA analysis discussed in the next sections.

First, we computed the mean value of each feature within the 5-minute window of each collected feature sample, as follows

1 $$\begin{aligned} f^i_{mean,k} = \frac{1}{N_k} \sum _{n=1}^{N_k}{f^i_{k,n}} \end{aligned}$$

where *i* is the index used to refer to each feature (e.g., *i* ranging from 1 to 20 identifies the 20 MFCC features, from 21 to 32 identifies the 12 Chroma features, and from 33 to 160 identifies the 128 Mel features), *k* is the index of the feature samples ranging from 1 to 275, and $N_k$ is the number of sentences captured within the 5-minute windows of the *k*-th sample.

Next, for each feature sample we computed the mean value of each type of speech feature, as follows

2 $$\begin{aligned} MFCC_{mean,k}= \frac{1}{20} \sum _{i=1}^{20}{\hat{f}^i_{mean,k}} \end{aligned}$$

3 $$\begin{aligned} Chroma_{mean,k}= \frac{1}{12} \sum _{i=21}^{32}{\hat{f}^i_{mean,k}} \end{aligned}$$

4 $$\begin{aligned} Mel_{mean,k}= \frac{1}{128} \sum _{i=33}^{160}{\hat{f}^i_{mean,k}} \end{aligned}$$

where $\hat{f}$ is the value of the feature after normalization, which was needed because different features (even belonging to the same type of speech feature) have different scales. The min-max normalization was applied by setting 0 and 1 as the new minimum and maximum values. In this way, we obtained a scalar value for each of the three considered types of speech features collected for each feature sample.

Finally, to compute the ‘All features’ value we refer in the next sections, we have computed the mean value of the three types of features for each feature sample.

### ACR scores

First, we computed the ANOVA test for the ACR scores and all the speech features extracted (combination of MFCCs, Chroma, and Mel features) for the three considered working activities, namely “Web Call”, “Talking at the office”, and “Talking at home”. ANOVA results, in terms of F-score and p-value, are shown in Table 1. The resulting high values of F-score, together with p-values $<0.001$ suggest significant changes of extracted speech features when different ACR scores are perceived, i.e., the means of the speech feature distributions related to different perceived QRWE (ACR score) are significantly different from each other. This result verifies for each of the 3 activities as well as when considering the ACR scores (and speech features) collected for all the working activities.

Moreover, Table 2 shows the results of the post-hoc pairwise multiple comparisons with Bonferroni correction. With regard to the “Web Call” activity, an adjusted p-value $\le 0.001$ has been obtained for all the 5 ACR scores. These results suggest that the means of the speech feature distributions, related to feedback containing different ACR scores, are significantly different among each other. Therefore, these set of features may be indicative of the QRWE perceived by the test participants. We have also found that the mean of the feature distribution for the ACR score ‘1’ is the most significantly different from the means of the feature distribution related to the other ACR scores. The utilization of the complete ACR scale by the participants in this case is justified by the fact that the Web Call was the most frequent activity for the conducted test.

With regard to the “Talking at the Office”, the ACR scale presents only the scores from 3 to 5. However, an adjusted p-value $\le 0.001$ has been obtained for all of these scores. Therefore, the means of the speech feature distributions, related to feedback containing different ACR scores, are significantly different among each other. We have also found that the mean of the feature distribution for the ACR score ‘5’ is the most significantly different from the means of the feature distribution related to the other ACR scores.

Finally, with regard to the “Talking at Home” activity, the ACR scale presents the scores from 2 to 5. An adjusted p-value $\le 0.001$ has been obtained for all of these scores except for the pair 3-4. Therefore, the means of the speech feature distributions, related to feedback containing ACR scores 3 and 4, are not significantly different between each other. This result prevents these ACR scores to be identified based on the observation of the speech features distribution. On the other hand, the mean of the feature distribution for the ACR score ‘5’ is the most significantly different from the means of the feature distribution related to the other ACR scores.

When considering all the activities, the pairwise comparison obtained an adjusted p-value $\le 0.001$ for all the 5 ACR scores, as for the “Web Call” activity. Note that these results are not in the Table for reasons of space. Thus, in case we do not distinguish among different working activities, we may observe the set of considered speech features as indicative of the QRWE perceived by the remote workers. We can then assume that the adjusted p-value $>0.001$ obtained for the “Talking at Home” activity for ACR scores 3 and 4 is likely due to the smaller number of samples collected.

In the following, we discuss the ANOVA results for each working activity by considering different types of speech features separately. The ANOVA results are summarized in Table 1, whereas the post-hoc pairwise multiple comparisons results are summarized in Tables 3, 4, and 5 for the MFCCs, Chroma, and Mel features, respectively.

**Table 2:** Post-hoc pairwise multiple comparison test with Bonferroni correction computed for ACR scores and all speech features for all the 3 working activities

ACR	Web Call					Talking at the Office			Talking at Home			
	1	2	3	4	5	3	4	5	2	3	4	5
1	–	$\le t$	$\le t$	$\le t$	$\le t$	–	–	–	–	–	–	–
2	$\le t$	–	$\le t$	$\le t$	$\le t$	–	–	–	–	$\le t$	$\le t$	$\le t$
3	$\le t$	$\le t$	–	$\le t$	$\le t$	–	$\le t$	$\le t$	$\le t$	–	$\mathbf >t$	$\le t$
4	$\le t$	$\le t$	$\le t$	–	$\le t$	$\le t$	–	$\le t$	$\le t$	$\mathbf >t$	–	$\le t$
5	$\le t$	$\le t$	$\le t$	$\le t$	–	$\le t$	$\le t$	–	$\le t$	$\le t$	$\le t$	–

$t=0.001$ is the threshold value for the adjusted p value. The cells containing the ﻿“-” symbol mean no p value was computed because: ACR score samples belonging to the same class (e.g., 1-1, 2-2, etc.); ACR scores were not provided for that working activity

**Table 3:** Post-hoc pairwise multiple comparison test with Bonferroni correction computed for ACR scores and the MFCC features for all the 3 working activities

ACR	Web Call					Talking at the Office			Talking at Home			
	1	2	3	4	5	3	4	5	2	3	4	5
1	–	$\le t$	$\le t$	$\le t$	$\le t$	–	–	–	–	–	–	–
2	$\le t$	–	$\mathbf >t$	$\mathbf >t$	$\le t$	–	–	–	–	$\mathbf >t$	$\mathbf >t$	$\le t$
3	$\le t$	$\mathbf >t$	–	$\mathbf >t$	$\le t$	–	$\le t$	$\le t$	$\mathbf >t$	–	$\mathbf >t$	$\le t$
4	$\le t$	$\mathbf >t$	$\mathbf >t$	–	$\le t$	$\le t$	–	$\mathbf >t$	$\mathbf >t$	$\mathbf >t$	–	$\le t$
5	$\le t$	$\le t$	$\le t$	$\le t$	–	$\le t$	$\mathbf >t$	–	$\le t$	$\le t$	$\le t$	–

$t=0.001$ is the threshold value for the adjusted p value. The cells containing the ﻿“-” symbol mean no p value was computed because: ACR score samples belonging to the same class (e.g., 1-1, 2-2, etc.); ACR scores were not provided for that working activity

**Table 4:** Post-hoc pairwise multiple comparison test with Bonferroni correction computed for ACR scores and the Chroma features for all the 3 working activities

ACR	Web Call					Talking at the Office			Talking at Home			
	1	2	3	4	5	3	4	5	2	3	4	5
1	–	$\le t$	$\le t$	$\le t$	$\le t$	–	–	–	–	–	–	–
2	$\le t$	–	$\mathbf >t$	$\mathbf >t$	$\le t$	–	–	–	–	$\mathbf >t$	$\mathbf >t$	$\le t$
3	$\le t$	$\mathbf >t$	–	$\mathbf >t$	$\le t$	–	$\le t$	$\le t$	$\mathbf >t$	–	$\mathbf >t$	$\le t$
4	$\le t$	$\mathbf >t$	$\mathbf >t$	–	$\mathbf >t$	$\le t$	–	$\mathbf >t$	$\mathbf >t$	$\mathbf >t$	–	$\le t$
5	$\le t$	$\le t$	$\le t$	$\mathbf >t$	–	$\le t$	$\mathbf >t$	–	$\le t$	$\le t$	$\le t$	–

$t=0.001$ is the threshold value for the adjusted p value. The cells containing the ﻿“-” symbol mean no p value was computed because: ACR score samples belonging to the same class (e.g., 1-1, 2-2, etc.); ACR scores were not provided for that working activity

**Table 5:** Post-hoc pairwise multiple comparison test with Bonferroni correction computed for ACR scores and the Mel features for all the 3 working activities

ACR	Web Call					Talking at the Office			Talking at Home			
	1	2	3	4	5	3	4	5	2	3	4	5
1	–	$\le t$	$\le t$	$\le t$	$\le t$	–	–	–	–	–	–	–
2	$\le t$	–	$\le t$	$\le t$	$\le t$	–	–	–	–	$\le t$	$\le t$	$\le t$
3	$\le t$	$\le t$	–	$\mathbf >t$	$\le t$	–	$\le t$	$\le t$	$\le t$	–	$\mathbf >t$	$\le t$
4	$\le t$	$\le t$	$\mathbf >t$	–	$\le t$	$\le t$	–	$\le t$	$\le t$	$\mathbf >t$	–	$\le t$
5	$\le t$	$\le t$	$\le t$	$\le t$	–	$\le t$	$\le t$	–	$\le t$	$\le t$	$\le t$	–

$t=0.001$ is the threshold value for the adjusted p value. The cells containing the ﻿“-” symbol mean no p value was computed because: ACR score samples belonging to the same class (e.g., 1-1, 2-2, etc.); ACR scores were not provided for that working activity

#### Web Call

We computed the ANOVA test for the ACR scores and the different types of speech features for the “Web Call” activity.

- *MFCCs features-ACR*: ANOVA provides an F-score of 48.12 and a p-value $\le 0.001$, computed on the entire ACR quality scale. The pairwise comparison obtained an adjusted p-value $\le 0.001$ only for the extreme ACR scores 1 and 5, whereas the ACR scores 2, 3, and 4 have means not significantly different among each other.
- *Chroma features-ACR*: ANOVA provides an F-score of 23.24 and a p-value $\le 0.001$, computed on the entire ACR quality scale. The pairwise comparison obtained an adjusted p-value $\le 0.001$ only for the extreme ACR scores 1 and 5 (except between 5 and 4), whereas the ACR scores 2, 3, and 4 have means not significantly different among each other.
- *Mel features-ACR*: ANOVA provides an F-score of 193.44 and a p-value $\le 0.001$, computed on the entire ACR quality scale. The pairwise comparison obtained an adjusted p-value $\le 0.001$ for all ACR scores except for the pair 3-4, which have means not significantly different among each other. However, this p-value $=0.004$ and is very close to the threshold.

#### Talking at the Office

We computed the ANOVA test for the ACR scores and the different types of speech features for the “Talking at the Office” activity.

- *MFCC features-ACR*: ANOVA provides an F-score of 37.07 and a p-value $\le 0.001$, computed on the 3 greatest scores of the ACR quality scale. The pairwise comparison obtained an adjusted p-value $\le 0.001$ only for the score 3 whereas the pairs 4-5 have means not significantly different between each other.
- *Chroma features-ACR*: ANOVA provides an F-score of 39.47 and a p-value $\le 0.001$, computed on the 3 greatest scores of the ACR quality scale. The pairwise comparison obtained an adjusted p-value $\le 0.001$ only for the score 3 whereas the pairs 4-5 have means not significantly different between each other.
- *Mel features-ACR*: ANOVA provides an F-score of 97.70 and a p-value $\le 0.001$, computed on the 3 greatest scores of the ACR quality scale. The pairwise comparison obtained an adjusted p-value $\le 0.001$ for all three ACR scores, which have means significantly different among each other.

#### Talking at home

We computed the ANOVA test for the ACR scores and the different types of speech features for the “Talking at Home” activity.

- *MFCC features-ACR*: ANOVA provides an F-score of 10.02 and a p-value $\le 0.001$, computed on the 4 greatest scores of the ACR quality scale. The pairwise comparison obtained an adjusted p-value $\le 0.001$ only for the score 5, whereas the scores 2, 3, and 4 have means not significantly different among each other.
- *Chroma features-ACR*: ANOVA provides an F-score of 9.95 and a p-value $\le 0.001$, computed on the 4 greatest scores of the ACR quality scale. The pairwise comparison obtained an adjusted p-value $\le 0.001$ only for the score 5, whereas the scores 2, 3, and 4 have means not significantly different among each other.
- *Mel features-ACR*: ANOVA provides an F-score of 57.78 and a p-value $\le 0.001$, computed on the 4 greatest scores of the ACR quality scale. The pairwise comparison obtained an adjusted p-value $\le 0.001$ for the extreme scores 2 and 5, whereas the scores 3 and 4 have means not significantly different between each other.

**Table 6:** ANOVA test for sentiment polarity and speech features

Activity	All features		MFCC features		Chroma features		Mel features	
	*F* score	*p* value	*F* score	*p*- value	*F* score	*p* value	*F* score	*p* value
Web Call	24.57	$< 0.001$	4.89	0.007	2.52	0.08	27.49	$< 0.001$
Talking at office	13.75	$< 0.001$	8.01	0.0048	7.78	0.0056	19.56	$< 0.001$
Talking at home	80.79	$< 0.001$	19.01	$< 0.001$	22.96	$< 0.001$	100.62	$< 0.001$
All activities	45.11	$< 0.001$	8.03	$< 0.001$	3.63	0.027	53.94	$< 0.001$

### Sentiment polarity

**Fig. 5:**
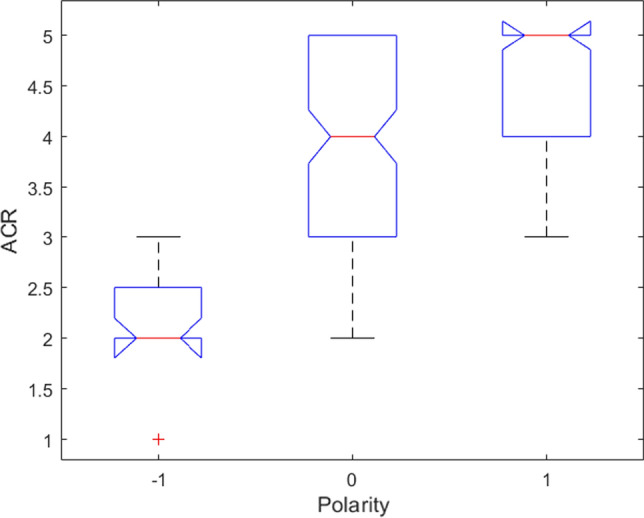
Box plot for the subjective impression (ACR scores - sentiment polarity)

In this section, we investigate the relationship between the perceived sentiment polarity and the QRWE as well as between the sentiment polarity and the collected speech features. The box plot in Fig. 5 indicates that the perceived QRWE and sentiment polarity are strongly related. In particular, positive quality perceptions are related to neutral and positive polarity whereas low quality is related to negative emotions. We then computed the Pearson correlation coefficient (PCC) and the Spearman’s rank correlation coefficient (SRCC) between the ACR scores and the polarity scores, whose value is 0.826 and 0.820, respectively. These results confirms the existing correlation between the perceived quality and the emotional feelings of the workers.

**Table 7:** Post-hoc pairwise multiple comparison test with Bonferroni correction computed for sentiment polarity and all speech features for all the 3 working activities

Polarity	Web Call			Talking at the Office			Talking at Home		
	− 1	0	1	− 1	0	1	− 1	0	1
− 1	–	$\le t$	$\le t$	–	–	–	–	$\le t$	$\le t$
0	$\le t$	–	$\mathbf >t$	–	–	$\le t$	$\le t$	–	$\le t$
1	$\le t$	$\mathbf >t$	–	–	$\le t$	–	$\le t$	$\le t$	–

$t=0.001$ is the threshold value for the adjusted p value. The cells containing the ﻿“−” symbol mean no p value was computed because: polarity belonging to the same class (e.g., 1-1, 0-0, etc.); polarity scores were not provided for that working activity

**Table 8:** Post-hoc pairwise multiple comparison test with Bonferroni correction computed for sentiment polarity and the MFCCs features for all the 3 working activities

Polarity	Web Call			Talking at the Office			Talking at Home		
	− 1	0	1	− 1	0	1	− 1	0	1
− 1	–	$\mathbf >t$	$\mathbf >t$	–	–	–	–	$\mathbf >t$	$\le t$
0	$\mathbf >t$	–	$\mathbf >t$	–	–	$\mathbf >t$	$\mathbf >t$	–	$\le t$
1	$\mathbf >t$	$\mathbf >t$	–	–	$\mathbf >t$	–	$\le t$	$\le t$	–

$t=0.001$ is the threshold value for the adjusted p-value. The cells containing the ﻿“-” symbol mean no p-value was computed because: polarity belonging to the same class (e.g., 1-1, 0-0, etc.); polarity scores were not provided for that working activity

**Table 9:** Post-hoc pairwise multiple comparison test with Bonferroni correction computed for sentiment polarity and the Chroma features for all the 3 working activities

Polarity	Web Call			Talking at the Office			Talking at Home		
	– 1	0	1	– 1	0	1	– 1	0	1
– 1	–	$\mathbf >t$	$\mathbf >t$	–	–	–	–	$\mathbf >t$	$\le t$
0	$\mathbf >t$	–	$\mathbf >t$	–	–	$\mathbf >t$	$\mathbf >t$	–	$\le t$
1	$\mathbf >t$	$\mathbf >t$	–	–	$\mathbf >t$	–	$\le t$	$\le t$	–

$t=0.001$ is the threshold value for the adjusted p-value. The cells containing the ﻿“-” symbol mean no p-value was computed because: polarity belonging to the same class (e.g., 1-1, 0-0, etc.); polarity scores were not provided for that working activity

**Table 10:** Post-hoc pairwise multiple comparison test with Bonferroni correction computed for sentiment polarity and the Mel features for all the 3 working activities

Polarity	Web Call			Talking at the Office			Talking at Home		
	– 1	0	1	– 1	0	1	– 1	0	1
– 1	–	$\le t$	$\le t$	–	–	–	–	$\mathbf >t$	$\le t$
0	$\le t$	–	$\mathbf >t$	–	–	$\le t$	$\mathbf >t$	–	$\le t$
1	$\le t$	$\mathbf >t$	–	–	$\le t$	–	$\le t$	$\le t$	–

$t=0.001$ is the threshold value for the adjusted p value. The cells containing the ﻿“-” symbol mean no p value was computed because: polarity belonging to the same class (e.g., 1-1, 0-0, etc.); polarity scores were not provided for that working activity

Therefore, we computed the ANOVA test for the sentiment polarity and all the speech features (combination of MFCCs, Chroma, and Mel features), whose results are summarized in Table 6. The resulting high values of F-score, together with p-values $<0.001$ suggest significant changes of extracted speech features when different sentiment polarities are perceived, i.e., the means of the distribution of the speech feature distributions related to different perceived polarity are significantly different from each other. This result verifies for each of the 3 activities as well as when considering the sentiment polarity (and speech features) collected for all the working activities. Moreover, Table 7 shows the results of the post-hoc pairwise multiple comparisons with Bonferroni correction. The pairwise comparison for “Web Call” obtained an adjusted p-value $\le 0.001$ for the negative sentiment whereas the neutral and positive sentiment have means not significantly different between each other. For “Talking at the Office” the adjusted p-value $\le 0.001$ for the neutral and positive sentiments (no negative sentiment in this case was perceived). Finally, for “Talking at Home” the adjusted p-value $\le 0.001$ for all the three sentiment polarities, which have means significantly different among each other. We have also found that when considering all the activities, the pair-wise comparison obtained an adjusted p-value $\le 0.001$ for all the three sentiment polarities, as for the “Talking at Home” activity.

In the following, we discuss the ANOVA results for each working activity by considering different types of speech features separately. The ANOVA results are summarized in Table 6, whereas the post-hoc pairwise multiple comparisons results are summarized in Tables 8, 9, and 10 for the MFCCs, Chroma, and Mel features, respectively.

#### Web Call

We computed the ANOVA test for the sentiment polarity and the different types of speech features for the “Web Call” activity.

- *MFCC features-ACR*: ANOVA provides an F-score of 4.89 and a p-value $> 0.001$, computed on the three polarities. The pairwise comparison obtained an adjusted p-value $>0.001$ for all the three polarities which have means not significantly different among each other.
- *Chroma features-ACR*: ANOVA provides an F-score of 2.52 and a p-value $> 0.001$, computed on the three polarity scores. The pairwise comparison obtained an adjusted p-value $>0.001$ for all the three polarities which have means not significantly different among each other.
- *Mel features-Polarity*: ANOVA provides an F-score of 27.49 and a p-value $\le 0.001$, computed on the three polarity scores. The pairwise comparison obtained an adjusted p-value $\le 0.001$ for the negative polarity whereas neutral and positive polarities have means not significantly different between each other.

#### Talking at the Office

We computed the ANOVA test for the sentiment polarity and the different types of speech features for the “Talking at the Office” activity.

- *MFCC features-ACR*: ANOVA provides an F-score of 8.01 and a p-value $> 0.001$, computed on the neutral and positive polarities. The pairwise comparison obtained an adjusted p-value $> 0.001$ for these polarities, which have means not significantly different between each other.
- *Chroma features-ACR*: ANOVA provides an F-score of 7.78 and a p-value $> 0.001$, computed on the neutral and positive polarities. The pairwise comparison obtained an adjusted p-value $> 0.001$ for these polarities, which have means not significantly different between each other.
- *Mel features-Polarity*: ANOVA provides an F-score of 19.56 and a p-value $\le 0.001$, computed on the neutral and positive polarities. The pairwise comparison obtained an adjusted p-value $\le 0.001$ for for these polarities, which have means significantly different between each other.

#### Talking at home

We computed the ANOVA test for the sentiment polarity and the different types of speech features for the “Talking at the Home” activity.

- *MFCC features-ACR*: ANOVA provides an F-score of 19.01 and a p-value $\le 0.001$, computed on the three polarity scores. The pairwise comparison obtained an adjusted p-value $\le 0.001$ for the positive polarity whereas the neutral and negative have means not significantly different between each other.
- *Chroma features-ACR*: ANOVA provides an F-score of 22.96 and a p-value $\le 0.001$, computed on the three polarity scores. The pairwise comparison obtained an adjusted p-value $\le 0.001$ for the positive polarity whereas the neutral and negative have means not significantly different between each other.
- *Mel features-Polarity*: ANOVA provides an F-score of 100.62 and a p-value $\le 0.001$, computed on the three polarity scores. The pairwise comparison obtained an adjusted p-value $\le 0.001$ for for the positive polarity whereas the neutral and negative have means not significantly different between each other. However, in this case the p-value $= 0.003$, which is very close to the threshold.

## Discussion

The conducted experiments have highlighted interesting findings regarding the QRWE and sentiment polarity perceived by remote workers. First of all, it is found that the “Web Call” was the most frequent activity conducted by the involved test participants during their remote working hours, followed by “Talking at the office” and “Talking at home”. This result confirms our expectation that most of the time spent during remote working involves participating in audio-video calls. In particular, the number of feedback provided for “Web Call” was almost 3 times that of “Talking at the office” and more than 4 times that of “Talking at home”.

Despite we have not investigated on the influence factors that driven the remote workers to provide their feedback regarding the perceived QRWE, we were able to identify some interesting differences among the three considered working activities. In particular, as shown in Fig. 3, the participants used the complete ACR scale only for the “Web Call” whereas the greatest 4 values of the scale (2-5) and the greatest 3 (3-5) were used respectively for the “Talking at home” and “Talking at the office” activities. This result indicates that the “Web Call” was the most annoying remote working task, leading the participants to rate the QRWE even with the lowest quality rates, although with minor frequency. On the contrary, the scores concerning “Talking at home” and “Talking at the office” suggest that having a physical talk with colleagues or relatives increases the perception of the working quality. Indeed, spending some time talking with other people in presence may be beneficial to make a small break from the working activities. However, it must be also considered that the data sample collected for these two activities was smaller than that collected for the Web calls; thus, the comparison is not balanced to draw precise conclusions but further data needs to be collected.

The ANOVA analysis regarding ACR scores (QRWE) and the collected speech features has provided interesting results. When considering all the speech features (i.e., a combination of MFCCs, Chroma and Mel features), the ANOVA results suggest significant changes of speech features when different ACR scores are perceived, i.e., the means of the speech feature distributions related to different QRWE (ACR score) are significantly different from each other. The only exception regards the speech features related to the ACR scores 3 and 4 rated for the “Talking at home” activity. However, this may be likely due to the smallest sample data collected for this activity. Therefore, these results indicate that there could be the possibility to train a ML-based model using the collected speech features to predict the QRWE perceived by the remote worker in terms of ACR scores. This result may be explained by the significance of the collected features, which are among the most relevant features used to represent spectral and acoustic speech characteristics in emotion detection tasks.

In section “ACR scores”, we have also conducted further ANOVA analysis by considering different types of speech features separately. It resulted that the consideration of a singular type of speech feature still permits to distinguish among the three considered working activities (see Table 1). However, the post-hoc pairwise multiple comparison (see Tables 3, 4, and 5 for MFCCs, Chroma, and Mel features, respectively) shows that the MFCCs alone, and the Chroma alone, are not different enough to be used to predict different QRWE perceived by the workers. These two types of features achieve good results (have means significantly different) only when related to extreme values of ACR scores, e.g., may be used to predict between poor or excellent quality, which can still be useful in quality assessment. On the other hand, the Mel features alone achieve results very close to those achieved when considering all features together, which however remains the best choice. This result indicates that the MFCCs and Chroma features represent some information that the Mel features are not able to capture.

With regard to the sentiment polarity, we have found that it is strongly correlated with the perceived QRWE. In particular, great quality perceptions (ACR from 3 to 5) are related to neutral and positive polarity whereas low quality perceptions (ACR from 1 to 2) are related to negative emotions. The absence of a natural interaction (Web Call) could then even affect the polarity of the worker with negative sentiments due to low quality perception. Therefore, we can state that the perception of a good QRWE has an important effect even on the positive emotional state of the worker, which in turn may have an impact on his productivity.

The ANOVA analysis regarding sentiment polarity and all the collected speech features (i.e., a combination of MFCCs, Chroma and Mel features) shows that to different sentiment polarities correspond different distributions for the speech features, such as to assume means significantly different among each other. Therefore, these speech features may be potentially observed to infer both the QRWE and the sentiment polarity of the speaker. However, the same results are not obtained in this case when considering singular type of speech features, since only the Mel features alone permit to distinguish among the three considered working activities (see Table 6). The post-hoc pairwise multiple comparison confirm these results (see Tables 8, 9, and 10 for MFCCs, Chroma, and Mel features, respectively) suggesting that the Mel features alone provide better results than MFCCs and Chroma features alone. However, the combination of all the features remains the best choice to predict between the perceived sentiment polarities.

Based on the obtained results, it is not daring to state that there could be the possibility to create a model, based on ML techniques, for estimating the perceived QRWE and polarity based on all the observed speech features. However, this would need more data and, especially, more balanced data. Indeed, the data collected in this study was unbalanced as the “Web Call” activity was far more frequent than the other two. Also, except for the “Web Call” activity, the ACR scale and polarity scale were not totally used by workers for rating, which is a problem for the training of an estimator.

## Conclusion

We conducted an analysis to investigate the QRWE (and sentiment polarity) of workers when carrying on different remote working activities. The ANOVA analysis was used to study the relationship between the perceived QRWE and the implicit emotion responses estimated from the speech of workers recorded during the conducted activities. ANOVA results suggest that speech features may be potentially observed to infer the QRWE and the sentiment polarity of the speaker. Therefore, there could be the possibility to create a ML-based model for estimating the perceived QoE and polarity based on the observed speech features. However, the training of such an estimator would need more data, and specifically, more balanced data. Moreover, it is also important to note that the subjects involved in the study were all linked with the University of Cagliari (even if with different roles, from PhD student to secretary), which may have introduced some biases. These aspects should be considered in future tests.

Further studies are also needed to investigate the influence factors impacting on the QRWE. For example, the network traffic could be monitored to identify slow Internet connections and its influence on the quality; also, remote workers could be asked to provide the reasons regarding negative feedback so as to identify which factors impact the most on the QRWE.

## Footnotes

1 https://github.com/alphacep/vosk-api.
